# Modeling and Vibration Control of Sandwich Composite Plates

**DOI:** 10.3390/ma16030896

**Published:** 2023-01-17

**Authors:** Zhicheng Huang, Huanyou Peng, Xingguo Wang, Fulei Chu

**Affiliations:** 1 College of Mechanical and Electrical Engineering, Jingdezhen Ceramic University, Jingdezhen 333001, China; 2 Department of Mechanical Engineering, Tsinghua University, Beijing 100084, China

**Keywords:** sandwich composites, finite element modeling, model order reduction, LQR controller, parameter optimization

## Abstract

A finite element dynamic model of the sandwich composite plate was developed based on classical laminate theory and Hamilton’s principle. A 4-node, 7-degree-of-freedom three-layer plate cell is constructed to simulate the interaction between the substrate, the viscoelastic damping layer, and the piezoelectric material layer. Among them, the viscoelastic layer is referred to as the complex constant shear modulus model, and the equivalent Rayleigh damping is introduced to represent the damping of the substrate. The established dynamics model has too many degrees of freedom, and the obtained dynamics model has good controllability and observability after adopting the joint reduced-order method of dynamic condensation in physical space and equilibrium in state space. The optimal quadratic (LQR) controller is designed for the active control of the sandwich panel, and the parameters of the controller parameters, the thickness of the viscoelastic layer, and the optimal covering position of the sandwich panel are optimized through simulation analysis. The results show that the finite element model established in this paper is still valid under different boundary conditions and different covering methods, and the model can still accurately and reliably represent the dynamic characteristics of the original system after using the joint step-down method. Under different excitation signals and different boundary conditions, the LQR control can effectively suppress the vibration of the sandwich plate. The optimal cover position of the sandwich plate is near the solid support end and far from the free-degree end. The parameters of controller parameters and viscoelastic layer thickness are optimized from several angles, respectively, and a reasonable optimization scheme can be selected according to the actual requirements.

## 1. Introduction

**Figure 1:**
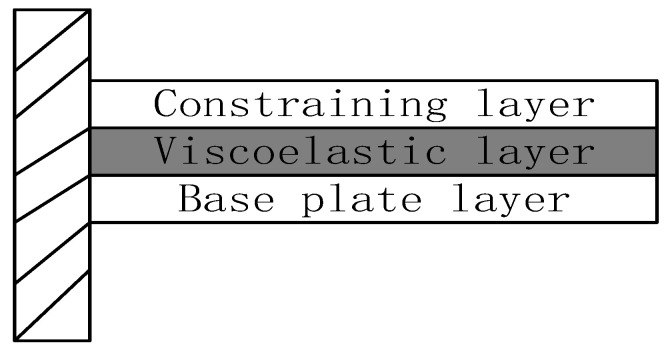
Constrained damping sandwich panels.

**Figure 2:**
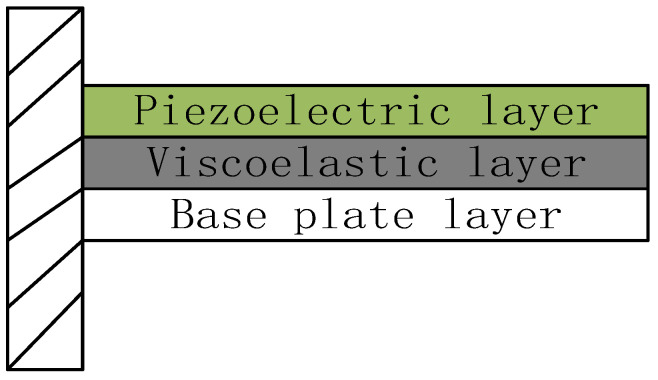
Active restrained damping sandwich panels.

With the development of the social economy and the improvement of people’s living standards, more and more industrial products have come into thousands of households, especially in recent years. The automotive industry is in the midst of rapid development. Many car-making enterprises use the MVH performance of the car as the technical highlight. In the design process will encounter the problem of excessive system vibration. The system structure vibration has become a hot issue in today’s society [1]. Classified from the control point of view, it can be divided into passive control, active control, and integrated active-passive control. For example, damping coatings, foam materials, constrained layer damping structures, composite damping structures, and ABA thermal wall liners. All belong to passive control methods [2], and Figure 1 shows a typical constrained damping sandwich panel structure. The mechanical energy generated by the structure is mainly transformed into strain energy and finally dissipated in the form of thermal energy through the damping layer in the middle [3]. This approach has the advantages of simple design, stable operation, and low cost, and it can effectively control the high-frequency vibration of the system. However, the shortcomings of this approach are that the damping provided is fixed, the control band is narrow, it lacks flexibility, and the control effect is limited in the low-frequency range [4]. The emergence of active control compensates for this deficiency by using smart materials such as piezoelectric ceramics as piezoelectric layers pasted on the surface, which can achieve the mutual conversion of electrical and kinetic energy through the piezoelectric effect, effectively achieving control of low-frequency vibrations [5]. However, the shortcomings of this approach are limited damping characteristics, high cost, major system failure when the controller fails, low stability, and limited effect on vibrations in the high-frequency range [6]. Therefore, integrated active and passive control can combine the advantages of the first two control methods, and the disadvantages compensate for each other [7]. Figure 2 shows a typical active restrained damping sandwich panel structure, which combines passive damping and active control [8].and differs from Figure 1 by replacing the uppermost restrained layer with a piezoelectric material layer. By applying piezoelectric forces to the structure through the piezoelectric ceramic material, the system can be effectively controlled in the low-frequency range, and the damping in the middle can further reduce the cost and improve the stability of the system. This approach is characterized by a simple structure, fast response, small additional mass, and wide controllable frequency domain [9].

Establishing a mathematical model of the structure is the premise of research control. At present, common methods include the analytical method, numerical method, and experimental method. Analytical methods can be used to establish the dynamic equation of a simple structure, including geometry, material, and other parameters. In the face of a complex structure, a higher-order differential equation will be generated. Generally, the Galerkin method or mode method is needed to solve the equation approximately. There are many shortcomings in practical application [10]. The numerical method can solve some complex sandwich structure models. Common numerical methods include the Ritz method, Galerkin method, Finite Element Method (FEM), and Spectral Finite Element Method (SFEM). The Ritz method and Galerkin method are very dependent on the choice of basis functions and are not suitable for complex boundary conditions. When the SFEM method is applied to a complex sandwich composite structure, the characteristic equation is complicated, the order is too high, and the parameters are unknown, so it is quite difficult to obtain the dynamic shape function [11]. The finite element method (FEM) is a well-known and highly effective technique for the computation of approximate solutions to complex and boundary value problems. Among the approximate solution methods, the FEM follows a systematic way by dividing the system into parts to solve complex problems. It gives the opportunity to easily take into account the support conditions, the continuous and sudden changes of external effects, and irregular geometries. The FEM method is very suitable for the modeling of the sandwich composite plate structure in this paper. The experimental method is used to verify the accuracy of the above two methods and is generally not used separately in engineering modeling [12]. On the other hand, the modeling characteristics of viscoelastic materials (VEM) should also be considered when using FEM sandwich composite panels for modeling, among which the common models are: the complex constant shear modulus model, ADF model, and GHM model. The degree of freedom of the mathematical model obtained through FEM modeling is too large for the subsequent active control work, so the model needs to be downscaled, and different downscaling methods have been proposed by domestic and foreign scholars, mainly used in conjunction with the two disciplines of finite element analysis and control theory. The common methods can be divided into two categories: step-down in physical space and step-down in state space. For sandwich composite plate structures, common control methods include proportional-integral differential (PID) control, linear quadratic optimal control, independent modal space control, robust control, etc. Baz [13] used the spectral transfer matrix method (STMM) and the spectral finite element method (SFEM) to model the ACLD sandwich beam, respectively, solved the exact solutions of the two methods, and discussed their advantages and disadvantages. Liao [14] established the model of EACLD beam structure by the Ritz method, characterized viscoelastic materials by the GHM model, and obtained discrete equations by the Galerkin method. The boundary conditions are simply supported at both ends. Shen [15] studied the control effect of the first and second bending vibration modes of ACLD sandwich plates using the Galerkin method. Huang [16] obtained the constrained damped sandwich plate dynamics equations based on first-order shear deformation and Hamilton’s principle and discussed the effects of layer thickness and loss factor of viscoelastic material on the inherent characteristics of the system. Zhang [17] developed an active control model for the stern bearing structure and designed an adaptive controller using the FXLMS algorithm. Huang and Mao [18] investigated the optimal coverage position of the piezoelectric sheet for the piezoelectric smart sandwich plate and the intelligent control using velocity feedback control. Cao [19] used classical plate theory to derive the control equations for a dual piezoelectric composite damping plate and compared the intrinsic characteristics and displacement parameters of the system with and without the control voltage.LI [20] established an ACLD cantilever beam dynamics model by experimental method and used different excitation signals to verify the reasonableness of the model. Because of the hysteresis effect of the viscoelastic layer, the obtained phase values were small. Lu [21] et al. established the dynamics equations of mechanically constrained layer-damped thin plate structure by invoking the ADF damping model and the GHM damping model Cao Y Q [22] designed a controller using optimal control theory and adaptive feedforward filtering algorithm to perform theoretical simulation analysis of the SCLD sandwich plate structure. Zhang [23] designed a feedback LQG and feedforward FXLMS-based adaptive control composite controller to investigate the ACLD plate structure to simulate and analyze the vibration problem and compare the performance of the composite controller. Zheng [24] et al. used proportional differential (PD) control for closed-loop vibration control of ACLD plate and cylindrical shell.

It can be seen through the literature that there are more studies on composite sandwich structures, but there are also some shortcomings: (1) the problem of accuracy of composite sandwich panel modeling, whether it can adapt to the actual application scenario, and whether it can remain stable under different boundary conditions and different covering methods. The dimensionality of the model is too large, leading to the inconvenient design of the controller. (2) For the problem of parameter selection of the controller, the problem of optimizing the structural parameters of the sandwich panel, for example, the problem of the best-covering position of the sandwich panel when the system adopts the local covering method. To address the above deficiencies, this paper adopts the finite element method to mathematically model the sandwich composite panel structure; the complex constant shear modulus model is used to characterize the VEM material properties; the Rayleigh damping is used to express the base layer properties to derive the system dynamics equations. The controllable system is established by the joint reduced-order method of dynamic condensation in physical space and internal equilibrium in state space. The LQR controller is used to control the vibration of the sandwich panel, and the selection scheme of the controller parameters, the optimal covering position of the sandwich panel unit, and the optimization scheme of the viscoelastic layer thickness are derived through simulation analysis.

## 2. Finite Element Method Modeling

**Figure 3:**
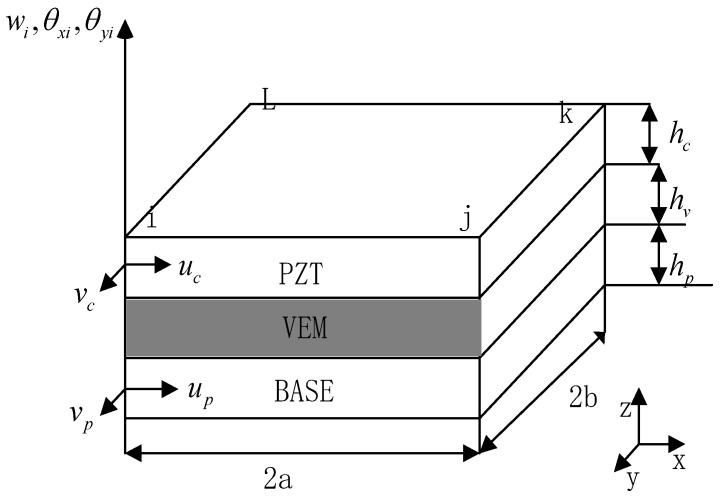
Schematic diagram of sandwich plate unit and local coordinates.

The sandwich panel is divided into several small cells, as shown in Figure 3. Each cell has four nodes, and each node has 7 degrees of freedom, respectively, x- and y-directional displacements in the piezoelectric material plane, x- and y-directional displacements in the neutral plane of the substrate, transverse displacements along the z-direction of the overall structure of the cell, and the unit’s turning angle around the x- and *y*-axis directions, which are expressed as $v_{c}$, $v_{c}$, $u_{p}$, $v_{p}$, w, $\theta_{x}$, $\theta_{y}$ so a rectangular cell has 28 degrees of freedom. The modeling using the finite element method satisfies the seven assumptions mentioned in the literature [25,26].

### 2.1. Unit Deformation and Motion Relationship

**Figure 4:**
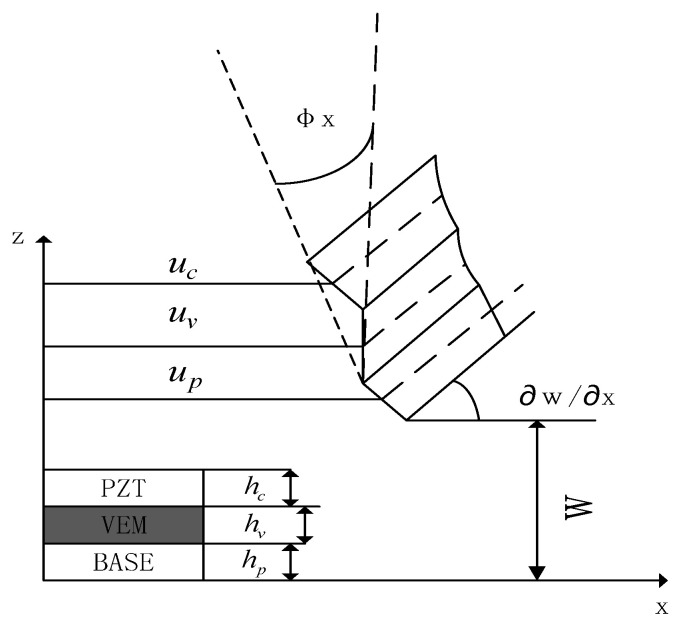
Deformation displacement of plate elements.

According to the assumptions and the analysis of unit degrees of freedom, Figure 4 shows the coupled geometric deformation relationship of the sandwich composite plate unit, the displacement of the viscoelastic material layer in the *x*-axis direction and *y*-axis direction are as follows [2,27]:

(1) $$u_{v}=\frac{1}{2}[(u_{c}+u_{p})+\frac{h_{c}-h_{p}}{2}\frac{\partial_{w}}{\partial_{x}}],v_{v}=\frac{1}{2}[(v_{c}+v_{p})+\frac{h_{c}-h_{p}}{2}\frac{\partial_{w}}{\partial_{x}}]$$

The shear strain resulting from the rotation of the viscoelastic layer in the x- and *y*-axis directions is:

(2) $$\beta_{x}=\frac{u_{c}-u_{p}}{h_{v}}+\frac{d}{h_{v}}\frac{\partial_{w}}{\partial_{x}},\beta_{y}=\frac{v_{c}-v_{p}}{h_{v}}+\frac{d}{h_{v}}\frac{\partial_{w}}{\partial_{x}}$$

where $u_{c},u_{v},u_{p}$ are the displacements of the neutral surfaces of the piezoelectric material layer, the viscoelastic layer, and the substrate layer in the x-direction, respectively, and the displacements of the neutral surfaces of the piezoelectric material layer, the viscoelastic layer and the substrate layer in the y-direction, respectively, and the thicknesses of the piezoelectric material layer, the viscoelastic layer, and the substrate layer, respectively [28]. Where $d=\frac{h_{c}+h_{p}}{2}+h_{v}$.

### 2.2. Unit Displacement Patterns and Form Functions

As shown in Figure 3, the composite plate unit is rectangular in shape, the dimensional length is 2a × 2b, each unit has 4 nodes (A, B, C, D), and each node has 7 degrees of freedom, assuming that the displacement vector of the node degrees of freedom is [25,29]:

(3) $$\{\Delta_{i}\}={\left[\begin{array}{ccccccc} u_{ci} & v_{ci} & u_{pi} & v_{pi} & w_{i} & \theta_{xi} & \theta_{yi} \end{array}\right]}^{T}$$

The displacement vector of the composite sandwich plates unit can be obtained as:

(4) $$\{U^{e}\}={\left\{\begin{array}{cccc} \Delta_{1} & \Delta_{2} & \Delta_{3} & \Delta_{4} \end{array}\right\}}^{T}$$

According to the nodal displacement pattern:

(5) $$u_{c}=a_{1}+a_{2}x+a_{3}y+a_{4}xy,\;v_{c}=a_{5}+a_{6}x+a_{7}y+a_{8}xy\;u_{p}=a_{9}+a_{10}x+a_{11}y+a_{12}xy,\;v_{p}=a_{13}+a_{14}x+a_{15}y+a_{16}xy\;w=a_{17}+a_{18}x+a_{19}y+a_{20}x^{2}+a_{21}xy+a_{22}y^{2}+a_{23}x^{3}+a_{24}x^{2}y+a_{25}xy^{2}+a_{26}y^{3}+a_{27}x^{3}y+a_{28}xy^{3}\;\theta_{x}=\frac{\partial_{w}}{\partial_{x}};\theta_{y}=-\frac{\partial_{w}}{\partial_{y}}$$

where $a_{1},a_{2},....a_{28}$ are determined by the 28 degrees of freedom displacement vectors of the four nodes of the unit. Therefore, the displacement position of any point within the composite sandwich plates unit can be derived from the interpolation of the displacement vector of the unit nodes as follows:

(6) $$\Delta ={\left[\begin{array}{ccccccc} u_{c} & v_{c} & u_{p} & v_{p} & w & \theta_{x} & \theta_{y} \end{array}\right]}^{T}=NU^{e}$$

where: $N={\left[\begin{array}{ccccccc} N_{1} & N_{2} & N_{3} & N_{4} & N_{5} & N_{6} & N_{7} \end{array}\right]}^{T}$ respectively correspond to the spatial interpolation vectors (shape functions) of $\begin{array}{ccccccc} u_{c} & v_{c} & u_{p} & v_{p} & w & \theta_{x} & \theta_{y} \end{array}$. Substitute the shape function matrix obtained above into Equations (1) and (2), and the shape function matrix of the viscoelastic layer can be obtained as follows:

(7) $$N_{8}=\frac{1}{2}[(N_{1}+N_{3})+\frac{h_{c}-h_{p}}{2}(-N_{7})],N_{9}=\frac{1}{2}[(N_{2}+N_{4})+\frac{h_{c}-h_{p}}{2}(N_{6})]$$

The shape function matrix of the shear strain of the viscoelastic layer is:

(8) $$N_{10}=\frac{1}{h_{v}}[(N_{1}-N_{3})-(\frac{h_{c}+h_{p}+2h_{v}}{2})(N_{7})],N_{11}=\frac{1}{h_{v}}[(N_{2}-N_{4})-(\frac{h_{c}+h_{p}+2h_{v}}{2})(N_{6})]$$

### 2.3. Finite Element Dynamics Equations

According to the classical thin plate theory, the kinetic energy, potential energy, and piezoelectric strain of each layer of the sandwich composite plate are derived by using the energy method and the principle of virtual work so that the mass matrix, stiffness matrix, and piezoelectric force matrix between each layer can be derived. The mass matrix of each layer of the sandwich plate unit is written directly [30,31].

The piezoelectric layer mass matrix is:

(9) $$M_{c}^{\left(e\right)}=p_{c}h_{c}\int_{0}^{2a}\int_{0}^{2b}\left(N_{1}^{T}N_{1}+N_{2}^{T}N_{2}+N_{5}^{T}N_{5}\right)dxdy$$

The viscoelastic layer mass matrix is:

(10) $$M_{v}^{\left(e\right)}=p_{v}h_{v}\int_{0}^{2a}\int_{0}^{2b}\left(N_{8}^{T}N_{8}+N_{9}^{T}N_{9}+N_{5}^{T}N_{5}\right)dxdy$$

The Base layer quality matrix is

(11) $$M_{p}^{\left(e\right)}=p_{p}h_{p}\int_{0}^{2a}\int_{0}^{2b}\left(N_{3}^{T}N_{3}+N_{4}^{T}N_{4}+N_{5}^{T}N_{5}\right)dxdy$$

where $p_{c},p_{v},p_{p}$ are the densities of the piezoelectric layer, viscoelastic layer, and Base layer, respectively. Then the sandwich composite panel structural unit mass matrix is:

(12) $$M^{\left(e\right)}=M_{c}^{\left(e\right)}+M_{v}^{\left(e\right)}+M_{p}^{\left(e\right)}$$

Write the stiffness matrix of each layer of the sandwich composite plates unit directly.

The piezoelectric layer stiffness matrix is:

(13) $$k_{c}^{\left(e\right)}=h_{c}\int_{0}^{2a}\int_{0}^{2b}B_{c}^{T}D_{c}B_{c}dxdy+\frac{h_{c}}{12}\int_{0}^{2a}\int_{0}^{2b}B^{T}D_{c}Bdxdy$$

The viscoelastic layer stiffness matrix is:

(14) $$k_{v}^{\left(e\right)}=h_{v}\int_{0}^{2a}\int_{0}^{2b}B_{v}^{T}D_{v}B_{v}dxdy+\frac{h_{v}}{12}\int_{0}^{2a}\int_{0}^{2b}B^{T}D_{v}Bdxdy$$

The shear stiffness matrix of the viscoelastic layer is:

(15) $$k_{\beta v}^{\left(e\right)}=Gh_{v}\int_{0}^{2a}\int_{0}^{2b}N_{10}^{T}N_{10}+N_{11}^{T}N_{11}dxdy$$

The Base layer stiffness matrix is:

(16) $$k_{p}^{\left(e\right)}=h_{p}\int_{0}^{2a}\int_{0}^{2b}B_{p}^{T}D_{p}B_{p}dxdy+\frac{h_{p}}{12}\int_{0}^{2a}\int_{0}^{2b}B^{T}D_{p}Bdxdy$$

where:

$$B=[N_{5},xx;N_{5}yy;2N_{5},xy],B_{c}=[N_{1},x;N_{2},y;N_{2},x+N_{1},y],B_{v}=[N_{8},x;N_{9},y;N_{8},x+N_{9},y],\;B_{p}=[N_{3},x;N_{4},y;N_{3},x+N_{4},y],D_{i}=\frac{E_{i}}{1-\mu_{i}^{2}}\left[\begin{array}{ccc} 1 & \mu_{i} & 0\\ \mu_{i} & 1 & 0\\ 0 & 0 & \frac{1-\mu_{i}}{2} \end{array}\right]$$

$B_{i}$ in ‘,’ denotes the derivative,

i=c, v, p.

where: $E_{c},E_{v},E_{p}$ are Young’s modulus of the piezoelectric layer, viscoelastic layer, and substrate layer, respectively, $\mu_{c},\mu_{v},\mu_{p}$ are Poisson’s ratio of the piezoelectric layer, viscoelastic layer, and Base layer, respectively. The stiffness matrix of the sandwich panel structural unit is:

(17) $$K^{e}=K_{c}^{e}+K_{v}^{e}+K_{p}^{e}+k_{\beta v}^{\left(e\right)}$$

The active control piezoelectricity matrix is:

(18) $$F_{c}^{\left(e\right)}=\frac{1}{2}V_{c}(t)\int_{0}^{2a}\int_{0}^{2b}B_{c}^{T}D_{c}{\left[\begin{array}{ccc} d_{11} & d_{22} & 0 \end{array}\right]}^{T}dxdy+\frac{1}{4}h_{c}V_{c}(t)\int_{0}^{2a}\int_{0}^{2b}B^{T}D_{c}{\left[\begin{array}{ccc} d_{11} & d_{22} & 0 \end{array}\right]}^{T}dxdy$$

where: $V_{c}^{e}$ is the value of applied voltage along the thickness direction of the piezoelectric layer, $d_{11}$ and $d_{22}$ are the piezoelectric constants.

The viscoelastic damping material is modeled with a complex constant shear modulus, so that G can be expressed as [32,33,34]:

(19) $$G=G_{V}(1+\eta i)$$

Considering that the Base has elastic damping, the proportional damping is used and expressed as:

(20) $$D=aM_{p}+bK_{p}$$

Using Hamilton’s principle, the kinetic equation of the sandwich composite plates unit can be derived as:

(21) $$M^{e}{\ddot{X}}^{e}+D^{e}{\dot{X}}^{e}+K^{e}X^{e}=F_{{}^{d}}^{e}+F_{c}^{e}$$

The total dynamic equation of the sandwich composite panel structure can be obtained by the conventional set of unit matrices and the introduction of boundary conditions as

(22) $$M\ddot{X}+D\dot{X}+KX=F_{d}+F_{c}$$

where: *M*, *D*, *K* is the total mass matrix, damping matrix, and stiffness matrix; $F_{d}$ and $F_{d}$ are the external excitation matrix and piezoelectric force matrix.

## 3. Model Downgrading

In order to facilitate the applicability of this model to various controller designs, this paper adopts a joint downscaling method of dynamical condensation in physical space and equilibrium in state space. The resulting system has greatly reduced degrees of freedom and is well-controllable and observable [30,31,35].

### 3.1. Dynamical Condensation in Physical Space

Rewrite Equation (22) in the following chunked matrix form:

(23) $$\left[\begin{array}{cc} M_{mm} & M_{ms}\\ M_{sm} & M_{ss} \end{array}\right]\left[\begin{array}{c} {\ddot{X}}_{m}\\ {\ddot{X}}_{s} \end{array}\right]+\left[\begin{array}{cc} D_{mm} & D_{ms}\\ D_{sm} & D_{ss} \end{array}\right]\left[\begin{array}{c} {\dot{X}}_{m}\\ {\dot{X}}_{s} \end{array}\right]+\left[\begin{array}{cc} K_{mm} & K_{ms}\\ K_{sm} & K_{ss} \end{array}\right]\left[\begin{array}{c} X_{m}\\ X_{s} \end{array}\right]=\left[\begin{array}{c} F_{cm}\\ F_{cs} \end{array}\right]$$

The subscripts m and s in the formula denote the primary and secondary degrees of freedom of the system, respectively. The dynamical condensation matrix between the primary and secondary degrees of freedom of the structure is:$R=K_{ss}^{-1}[(M_{sm}+M_{ss}R)M_{R}^{-1}K_{R}-K_{sm}]$, After (N) iterations the dynamical condensation matrix is:

$R^{N+1}=K_{ss}^{-1}[(M_{sm}+M_{ss}R^{N})(M_{R}^{N}{)}^{-1}K_{R}^{N}-K_{sm}]$, where the initial value of R is: $R^{0}=K_{ss}^{-1}K_{sm}$. Similar to *R*, the final condensation matrices $M_{R}$, $D_{R}$, $K_{R}$ can be obtained by iterating:

$$M_{R}^{N}=M_{mm}+{\left(R^{N}\right)}^{T}M_{sm}+{\left(R^{N}\right)}^{T}M_{ss}R^{N}+M_{ms}R^{N},\;D_{R}^{N}=D_{mm}+{\left(R^{N}\right)}^{T}D_{sm}+{\left(R^{N}\right)}^{T}D_{ss}R^{N}+D_{ms}R^{N}.\;K_{R}^{N}=K_{mm}+{\left(R^{N}\right)}^{T}K_{sm}+{\left(R^{N}\right)}^{T}K_{ss}R^{N}+K_{ms}R^{N},F_{CR}^{N}=F_{cm}+{\left(R^{N}\right)}^{T}F_{cs}.$$

In this paper, the X- and Y-directional displacements within the piezoelectric layer and Base level and the z-directional displacement of the structure in the sandwich composite plate structure are selected as the primary degrees of freedom, and the rest are selected as the second degrees of freedom. The final kinetic equations after the reduced order is:

(24) $$M_{R}^{N}{\ddot{X}}_{m}+D_{R}^{N}{\dot{X}}_{m}+K_{R}^{N}X_{m}=F_{CR}^{N}$$

### 3.2. Equilibrium Descending Order in State Space

Rewriting Equation (24) into the equation of state in state space is:

(25) $$\{\begin{array}{c} \overset{}{x=A\dot{x}+Bu}\\ y=Cx+Du \end{array}$$

where A, B, and C are denoted as the state matrix, input matrix, and input matrix of the system, respectively. The controllability Grammian matrix of the system is: $W_{c}=\int_{0}^{\infty }e_{A\tau }{}^{T}B^{T}Be_{A\tau },$ The Grammian matrix of system observability is: $W_{o}=\int_{0}^{\infty }e_{A\tau }{}^{T}C^{T}Ce_{A\tau }d\tau .$

In the above equation, when the controllability $W_{C}$ matrix of the system is full rank, it means that the system is controllable; when the observability matrix $W_{O}$ of the system is full rank, it means that the system is observable. Define a non-singular transformation matrix H and perform the equilibrium transformation: $\bar{x}=\mathrm{Hx}$ and substitute this transformation into Equation (25) to obtain the new controllability and observability matrices as:

(26) $$\bar{W_{c}}=HW_{c}H^{-1},\bar{W_{o}}=HW_{o}H^{-1}$$

When an appropriate similar transformation matrix *H* is chosen, $\bar{W_{c}}=\bar{W_{o}}=diag(g_{1},g_{2}\cdots g_{2n})$, such that an internal equilibrium change is achieved. At this point g has been sorted from large to small, and g indicates the observable and controllable index of the system modality. Therefore, the smaller weakly controllable and weakly observable values are removed so that the model can be downgraded, and the following is the downgrading process. The state variable of the new system after the transformation is expressed as:$\;\bar{x}$=${\left[{\bar{x}}_{r}\;{\bar{x}}_{d}\right]}^{T}$,$\;{\bar{x}}_{r}$ is the retained quantity and ${\bar{x}}_{d}$ is the deleted quantity, at which point the state equation of the system is:

(27) $$\left[\begin{array}{c} \dot{{\bar{x}}_{r}}\\ \dot{{\bar{x}}_{d}} \end{array}\right]=\left[\begin{array}{cc} \bar{A_{rr}} & \bar{A_{dr}}\\ \bar{A_{rd}} & \bar{A_{dr}} \end{array}\right]\left[\begin{array}{c} {\bar{x}}_{r}\\ {\bar{x}}_{d} \end{array}\right]+\left[\begin{array}{c} {\bar{B}}_{r}\\ {\bar{B}}_{d} \end{array}\right]u,\;y=[{\bar{C}}_{r}{\bar{C}}_{d}]\left[\begin{array}{c} {\bar{x}}_{r}\\ {\bar{x}}_{d} \end{array}\right]+\;\bar{D}u$$

Let ${\bar{x}}_{d}$ = 0, that is, remove the state variables, then the new equation of state after the equilibrium step-down can be obtained as:

(28) $$\{\begin{array}{c} \dot{{\bar{x}}_{r}}=\bar{A_{rr}}{\bar{x}}_{r}+{\bar{B}}_{r}u\\ y={\bar{C}}_{r}{\bar{x}}_{r}+\;\bar{D}u \end{array}$$

Equation (28) further reduces the model freedom dimension compared with Equation (25), and the model system is guaranteed to be observable and controllable.

## 4. Model Validation and Case Analysis

This section verifies the accuracy of the finite element model of the composite sandwich panel before and after the joint step-down, respectively. The optimum covering position of the piezoelectric sheet under different boundary conditions is derived from the arithmetic analysis, which provides a theoretical basis for the subsequent cell covering method during vibration control of the sandwich composite plates. The geometric and material parameters of each layer are as follows [21,36,37]:

Piezoelectric layer: $p_{c}=7450{\;\mathrm{kg}/m}^{3},E_{c}=74.5\;\mathrm{GPa},\mu_{c}=0.32,$ viscoelastic layer: $p_{V}=789{\;\mathrm{kg}/m}^{3},\mu_{V}=0.3,E_{v}=(2(1+u_{v}))G_{v}$.

$G_{V}=0.01(1+0.8i)\;\mathrm{Mpa};$ Base layer: $p_{p}=2800{\;\mathrm{kg}/m}^{3},E_{p}=70\;\mathrm{GPa},\mu_{p}=0.3,$ Size of the substrate: 0.20 m in full length and 0.10 m in width. $h_{c}=0.3\;\mathrm{mm},h_{v}=0.8\;\mathrm{mm},h_{p}=1\;\mathrm{mm}$. Piezoelectric constant: $d_{11}=d_{22}=2.8e-9$.

To verify the correctness of the finite element modeling method derived in this paper, the MATLAB programming of the equations derived in the paper was used to derive the numerical solutions and the results of the large-scale finite element analysis software ANSYS for comparison, and the model accuracy was further verified with the help of references and modal experimental results. As shown in Table 1, the inherent frequencies of the base plate were calculated under three boundary conditions. The boundary conditions of the four sides of the plate are represented by letters, and their meanings are C-fixed support and F-free. CFFF means that one side is fixed, and the other three sides are free: CFCF means that two opposite edges are fixed, and the other two edges are free. The size of the numerical solution of the intrinsic frequency derived from MATLAB software is similar to the ANSYS finite element results, as well as literature comparisons and experimental results, with the maximum error controlled within 5%. By changing the boundary conditions, the exact solution can still be calculated by the method in this paper.

**Figure 5:**
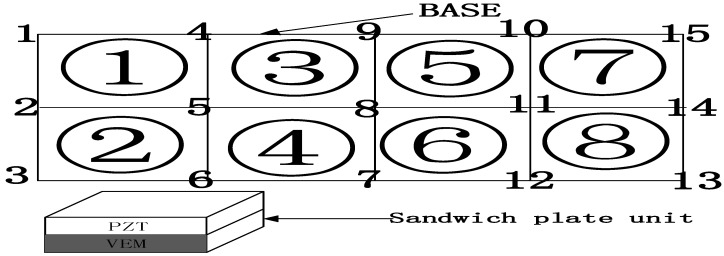
Sandwich plate unit covering method diagram.

As shown in Figure 5, the base layer is discrete into eight units with 15 nodes in total. (1–8) represents unit location, and (1–15) represents nodes location. The sandwich plate units are covered sequentially to derive the law of variation of the inherent characteristics of the system [38,39]. Thus, the optimal covering position of the piezoelectric sheet under different boundary conditions is found. As shown in Table 2, when the boundary condition is CFFF: the inherent frequency of the structure decreases gradually when the covered units increase, and the trend of decrease is faster and faster, and the frequency decrease is most obvious when the covered unit is (7.8). Therefore, when the boundary condition is CFFF, the best position of the piezoelectric sheet is the solid support end of the (1.2) cell, and the free end of the (7.8) cell should be avoided. When the boundary condition is CFCF: when covering the unit increases, the inherent frequency of the system gradually decreases, and the change of the inherent characteristics of the system is more obvious in the middle position (3–6 units), and the change of the decrease is smaller when covering (1.2.7.8 units). Therefore, when the boundary condition is CFCF, the best position of the piezoelectric sheet is the solid support end of (cell 1.2.7.8), and it avoids covering the middle position.

**Figure 6:**
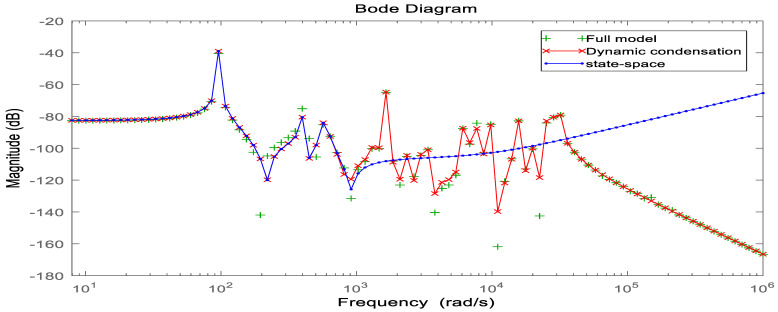
Bode diagram in the frequency domain before and after model reduction.

**Figure 7:**
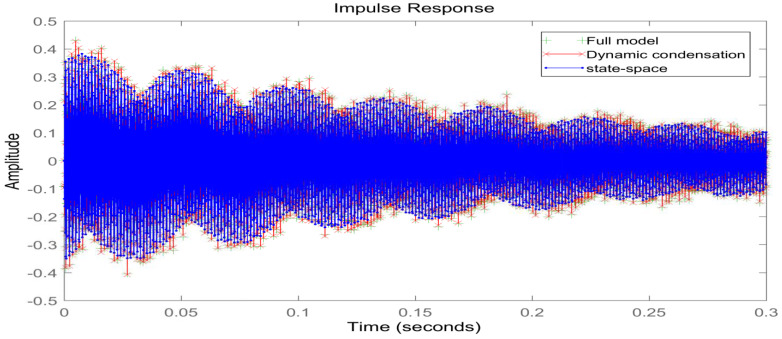
Time domain impulse response diagram before and after model reduction.

In order to verify the accuracy of the model after the joint downscaling method, the dynamic characteristics of the system before and after the downscaling process are compared, on the one hand, to analyze and compare the time-domain characteristics of the model before and after the downscaling, and on the other hand, to compare the frequency-domain characteristics of the model. As shown in Figure 6, it can be seen that the frequency response curve in the low-frequency mode range after the joint downscaling of the original system is basically the same as the frequency domain response curve of the original model. As shown in Figure 7, the time domain response of the original system under the impulse excitation after the two downscaling and the time domain response curve fitting degree shows that the time domain impact on the system after the two downscaling is small. As shown in Table 3, both the WC and WO matrices of the system are not full rank before the step-down, and the system is uncontrollable and uncontrollable, and after the step-down by equilibrium in the state space, both the WC and WO matrices are full rank, and the system reaches controllable and controllable. In summary, the proposed step-down approach in this paper is effective and not only reduces the system’s degrees of freedom significantly but also, the final system is considerable and controllable, which provides favorable help for the design of active controllers.

## 5. LQR Control and Simulation Analysis

When using LQR control, the reduced-order state-space equations of the system should be obtained, as in Equation (28) [40,41]. Design the optimal feedback controller such that the objective function *J* is minimized as:

(29) $${J=\int }_{0}^{\infty }\left(x_{r}^{T}Qx_{r}+u^{T}Ru\right)dt$$

where *Q* and *R* are the output vector weighting matrix, the control vector weighting matrix, and u is the control voltage of the system, respectively. $u=-kx_{r},k=R^{-1}{\bar{B}}_{r}{}^{T}P,$ *P* satisfies the Riccati equation: $P\bar{A_{rr}}+{\bar{A_{rr}}}^{T}p-p{\bar{B}}_{r}R^{-1}{\bar{B}}_{r}{}^{T}p+{\bar{C}}_{r}{}^{T}Q{\bar{C}}_{r}=0$ [42,43]. The corresponding closed-loop system equation of state is:

(30) $$\dot{x}=(\bar{A_{rr}}-{\bar{B}}_{r}K){\bar{x}}_{r}+{\bar{B}}_{r}u$$

### 5.1. Simulation of Vibration Control of Sandwich Composite Plates

In order to verify that the reduced-order model can be applied to the LQR controller, three signal excitations are used under the boundary conditions of CFFF and CFCF, respectively; namely, impulse excitation, band-limited white noise signal, and sinusoidal periodic signal. The sinusoidal period signal is determined by the first, second order intrinsic frequency value of the system. The control effect is judged by comparing the response curves in the time domain before and after the control.

**Figure 8:**
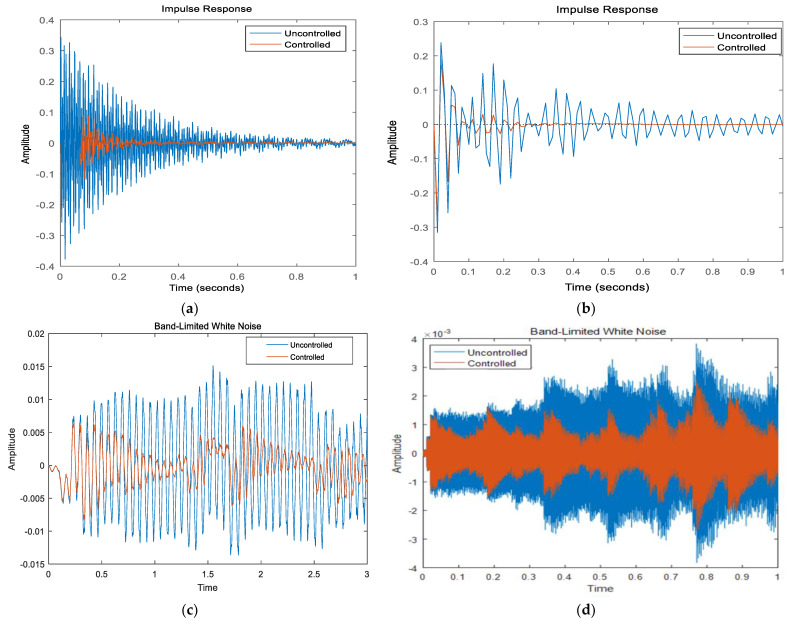

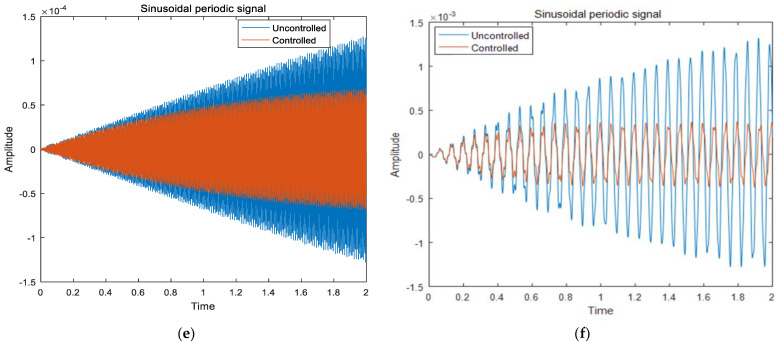
System control before and after response diagram. (**a**) CFFF Plate Response under Pulse Excitation; (**b**) CFCF Plate Response under Pulse Excitation; (**c**) CFFF plate response under band-limited white noise; (**d**) CFCF plate response under band-limited white noise; (**e**) CFFF plate response under sinusoidal period signal; (**f**) CFCF plate response under sinusoidal period signal.

As shown in Figure 8a,b: under the pulse signal excitation, the amplitude of the CFFF sandwich plate is reduced by about 62% before and after the application of the control voltage, and the convergence time is shortened from 1.8 s to about 0.6 s. The amplitude of the CFCF sandwich plate is reduced by about 68% before and after the application of the control voltage, and the convergence time is shortened from 3.4 s to about 0.5 s. As shown in Figure 8c,d: Under the excitation of the band-limited white noise signal, the RMS value of the displacement of the CFFF sandwich plate before and after the application of the control voltage is reduced by about 65%, and the maximum displacement is reduced from 1.5 mm to 0.5 mm. the RMS value of the displacement of the CFCF sandwich plate before, and after the application of the control voltage is reduced by about 73%, and the maximum displacement is reduced from 0.37 mm to 0.25 mm. as shown in Figure 8e,f The RMS value of the amplitude of the CFFF sandwich panel before, and after the application of the control voltage is reduced by about 49%, and the maximum displacement is reduced from 1.48 × 10^−4^ m to 0.51 × 10^−4^ m under the excitation of the sinusoidal periodic signal. The RMS value of the amplitude of the CFCF sandwich panel before and after the application of the control voltage is reduced by about 68%, and the maximum displacement is reduced from 1.42 × 10^−3^ m to 0.43 × 10^−3^ m. In summary, the LQR controller designed in this section can effectively control the composite sandwich plate structure, and it can maintain a stable and effective control effect under different boundary conditions and different excitation signals. The simulation basis is provided for the optimization of the controller parameters and the optimization of the parameters of the sandwich composite panels structure in the next section.

### 5.2. Controller Parameter Optimization

The fundamental point of the optimal controller design is to solve the Riccati equation, and the determination of the control gain matrix k is essentially the objective function J to reach the minimum. The values of Q and R weighting matrices in the equation have a great influence on the control effect of the structure, and it is worth studying and exploring how to determine the size and form of the Q and R matrices in the optimal controller. In this section, the form of the Q and R matrices is set as follows: Q is the diagonal matrix, and the optimal values of the Q and R matrices need to be derived by continuous trial and simulation, where are the coefficients of the matrices and I is the unit matrix. Simulation analysis is performed by changing the coefficients of the weighted matrix when the coefficients of the Q matrix change, R = I0. When the coefficients of the R matrix change, the coefficients of the Q matrix are 1 × 10^4^.

**Figure 9:**
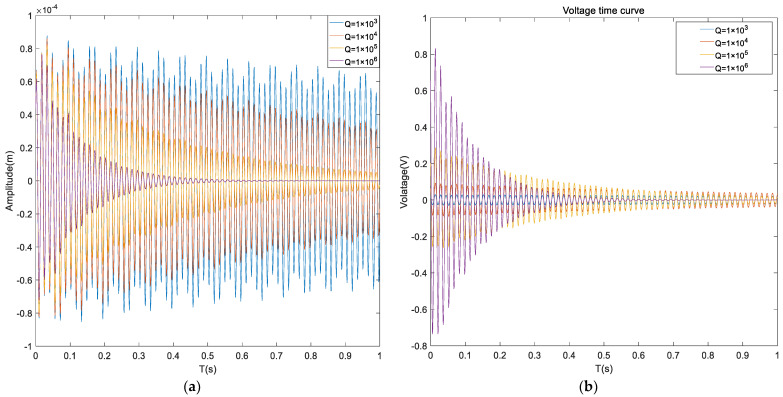
Comparison of system characteristics with different Q matrix coefficients. (**a**) Composite sandwich plates displacement time response; (**b**) Composite sandwich panel voltage-time curve.

**Figure 10:**
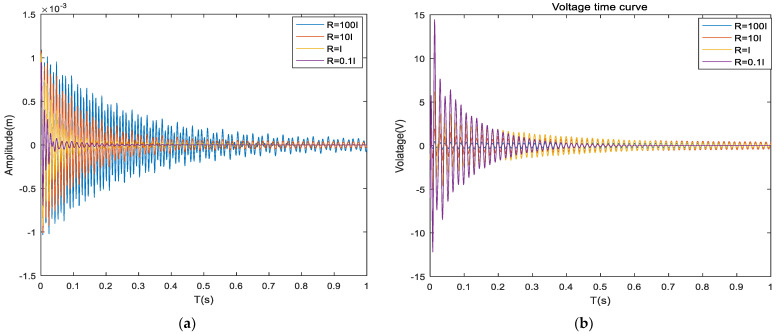
Comparison of system characteristics with different R matrix coefficients. (**a**) Composite sandwich plates displacement time response; (**b**) Composite sandwich panel voltage-time curve.

As shown in Figure 9, the maximum displacement is about 7.9 × 10^−4^ m with the LQR control with a Q matrix factor of 1 × 10^3^, the vibration convergence time is about 15 s, and the required maximum control voltage is about 0.3 V. The maximum displacement is about 7.78 × 10^−4^ m with the LQR control with a Q matrix factor of 1 × 10^4^, the vibration convergence time is about 9 s, and the required maximum control voltage is about 0.8 V. The maximum displacement is about 7.64 × 10^−4^ m with the LQR control with a Q matrix factor of 1 × 10^5^, and the required maximum control voltage is about 2.5 s. The maximum displacement is about 7.64× 10^−4^ m, the vibration convergence time is about 2.5 s, and the required maximum control voltage is about 2.3 V. The maximum displacement is about 6.62 × 10^−4^ m, the vibration convergence time is about 0.7 s, and the required maximum control voltage is about 8 V under LQR control with a Q matrix factor of 1× 10^6^. As shown in Figure 10, the maximum displacement is about 1.08 × 10^−3^ m with LQR control of R matrix factor 100, the vibration convergence time is about 2.5 s, and the required maximum control voltage is about 0.6 V. The maximum displacement is about 1.06 × 10^−3^ m with LQR control of R matrix factor 10, the vibration convergence time is about 2 s, and the required maximum control voltage is about 2.1 V. The maximum displacement is about 0.87 × 10^−3^ m, the vibration convergence time is about 1.4 s, and the required maximum control voltage is about 6.5 V. The maximum displacement is about 0.72 × 10^−3^ m, the vibration convergence time is about 0.4 s, and the required maximum control voltage is about 14 V under LQR control with an R matrix coefficient of 0.1.

In summary, the following conclusions can be found: (1) When the Q matrix coefficient increases, the amplitude convergence time of the system is greatly reduced, the required voltage value also increases, and the control effect is enhanced. (2) When the R matrix coefficient decreases, the amplitude convergence time of the system is greatly shortened, and the required voltage value also increases, which enhances the control effect. (3) The effect of changing Q and R matrix coefficients on the reduction of the maximum displacement of the sandwich composite plate is not strong, but the convergence time changes more obviously. This is also in line with the characteristics of the LQR controller. Changing the parameters of the controller alone is not the best optimization strategy, so it needs to be optimized from the structure.

### 5.3. Sandwich Composite Plates Cover Position Optimization

The optimal covering position of the composite sandwich panel is derived from the example analysis in Section 3. This section further verifies the conclusion by comparing the simulation analysis results with the results of the calculation example analysis. The coverage method in this section is different from the coverage method in the example analysis, which covers only two units at a time, the first coverage (units 1., 2), the second coverage (units 3, 4), and the third coverage (units 7, 8). Compare the response curves of the three coverage methods when the control voltage is not applied, the response curves of the sandwich laminate when the control voltage is applied, and the graph of the required voltage versus time.

**Figure 11:**
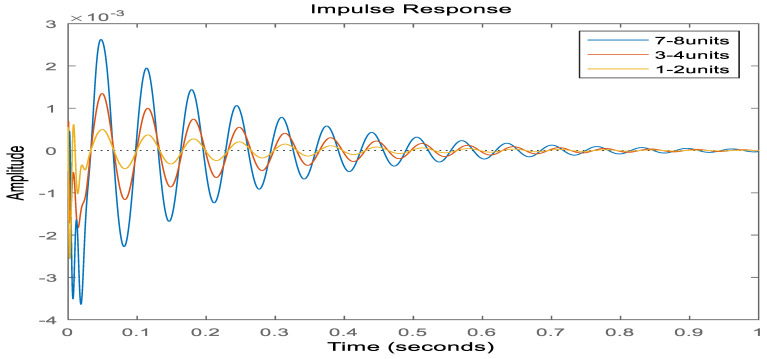
Response diagram of sandwich plates under different covering methods (without applied voltage).

**Figure 12:**
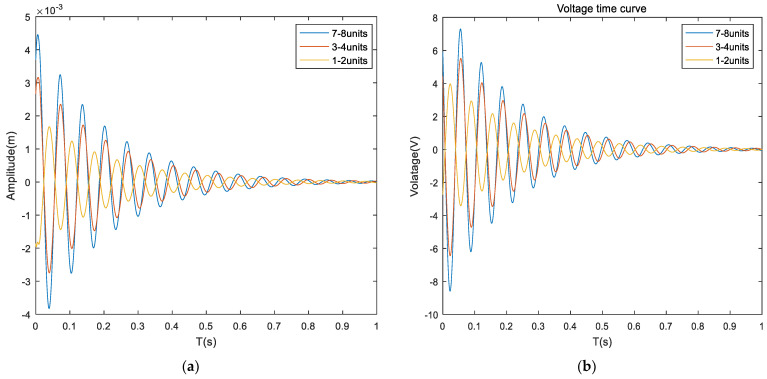
Response diagram of sandwich plates under different covering methods (applied voltage). (**a**) Composite sandwich plates displacement time response; (**b**) Composite sandwich panel voltage-time curve.

Figure 11 shows that the maximum amplitude of the system is 0.4 × 10^−3^ m, and the convergence time is 1.5 s when covering (1, 2) units without applying control voltage. When covering (3, 4) units, the maximum amplitude of the system is 1.3 × 10^−3^ m, and the convergence time is 1.6 s. When covering (7, 8) units, the maximum amplitude of the system is 2.6 × 10^−3^ m, and the convergence time is 1.8 s. Analysis of the data shows that: (1) When the covering position is different, the vibration amplitude of the system is not the same, which is because without applying the control voltage, the composite sandwich panel can suppress the structural vibration by passive control. (2) When the covering position is up to (1.2) unit, the maximum displacement reduction effect is obvious, but the convergence time effect is not obvious. Figure 12 shows: When the control voltage is applied, the system is given an initial displacement, and no excitation signal is applied. The maximum amplitude of the system is 1.7 × 10^−3^ m, and the convergence time is 1.4 s when covering (1, 2) cells. When covering (3, 4) cells, the maximum amplitude of the system is 3.1× 10^−3^ m, and the convergence time is 1.5 s. When covering (7, 8) cells, the maximum amplitude of the system is 4.3 × 10^−3^ m, and the convergence time is 1.8 s. After applying the control voltage, the parameters of the controller are the same, the amplitude is different for different coverage positions, and the active control effect is also different. (2) When covering (1, 2) units, the active control effect of the sandwich composite panel is the best, and the most important result is that the required voltage is also the least.

In summary: (1) The system vibration can be suppressed well by changing the unit coverage position, especially the maximum amplitude of the system can be reduced, but the convergence effect is not obvious. (2) For the sandwich composite plate with the boundary condition of CFFF, the best coverage is near the solid support end (1 and 2) units, and the conclusion of the simulation analysis is the same as the results of the calculation in Section 3. The optimal placement of the piezoelectric sheet by the modal vibration and modal strain energy method in the literature [23] and the position optimization based on the modal parametric number in the literature [15] are consistent with the results of the method in this paper.

### 5.4. Optimization of Viscoelastic Material Layer Parameters

The sandwich composite plate has active and passive integrated control functions. When the active controller fails, the passive control will play a role. Among them, the viscoelastic material layer plays a decisive role in the sandwich panel, and the parameters of the damping material determine the effect of passive control. In this section, the thickness of the viscoelastic material layer is investigated to compare the response curves when the control voltage is not applied at different thicknesses. And the response curves when the control voltage is applied at different thicknesses.

**Figure 13:**
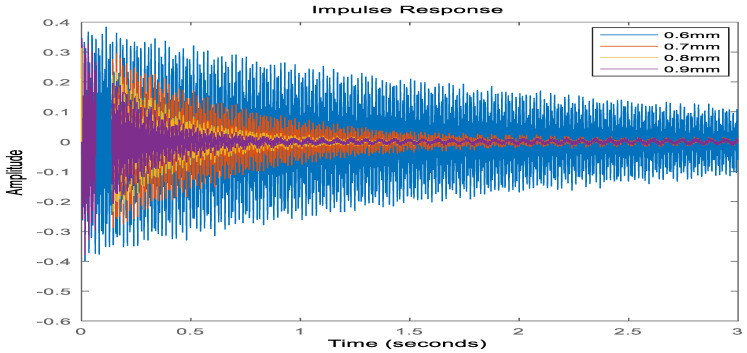
System response of viscoelastic material layer with different thickness (without applied voltage).

**Figure 14:**
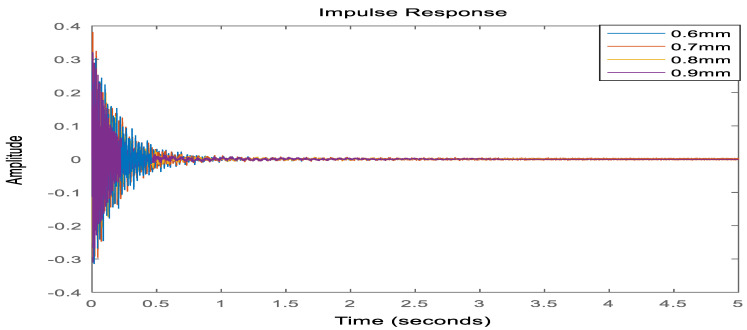
System response of viscoelastic material layer with different thickness (applied voltage).

Figure 13 shows that when the control voltage is not applied, and the pulse excitation signal is applied to the system, the damping layer can convert the kinetic energy into thermal energy to achieve passive control, and when the thickness of the damping layer increases, the vibration of the system can be effectively suppressed. The advantages and disadvantages of passive control by simply changing the thickness of the damping layer are as follows: the average amplitude value is reduced significantly, the system has a good suppression effect in the high-frequency vibration region, and the control cost is low. Disadvantages: from the point of view of convergence is not good. The system has been in a low-frequency vibration state. The control effect is limited, and increasing the thickness will lead to an excessive mass of the system. Figure 14 shows: apply a control voltage to the system, under the pulse signal excitation, the controller Q and R parameters are the same. At this time, the viscoelastic material layer in different thicknesses of the system response difference is not significant by changing the thickness of the damping layer way for active control effect is not obvious. In summary; (1) the stability of the composite sandwich panel can be enhanced by changing the thickness of the damping layer when the controller fails, but the effect is not proportional, as can be seen from Figure 13 when the thickness increases from 0.8 mm to 0.9 mm, the effect is not obvious. (2) The increase of the thickness of the damping layer does not improve the active control effect of the system, but on the contrary, the increase of the thickness leads to the overall mass of the structure being too large, so it is necessary to set the thickness of the damping layer reasonably.

## 6. Conclusions

In this paper, the dynamics model of sandwich composite panel structure under three different boundary conditions is established using the finite element method, where the covering method contains partially covered sandwich units and fully covered sandwich units. The accuracy of the model is improved by introducing the complex constant shear modulus model through the viscoelastic layer and the proportional damping through the base layer. The model suitable for the LQR controller can be obtained by the joint order reduction of the dynamic condensation under the physical space and the equilibrium method in the state space. The parameters of the LQR controller, the parameters of the viscoelastic material layer, and the optimal covering position of the sandwich panel are optimally designed by simulation analysis. The study shows that:

(1) The finite element model established in this paper is still valid under different boundary conditions and different covering methods. The introduction of the complex constant shear modulus damping model and the proportional damping of the base layer further improves the accuracy of the structural dynamics model. The mathematical model obtained by the joint reduced order method has low degrees of freedom, good controllability, and observability. The dynamical characteristics of the reduced-order model are essentially unchanged in both the time and frequency domains. It can be directly used for system controller design.
(2) Through simulation, it can be seen that the sandwich composite plates can effectively suppress the structural vibration with good adaptability under different boundary conditions and different excitation signals using the LQR controller. And the Q and R weighting matrix coefficients affect the system control effect, which provides a theoretical basis for further determining the optimal form and size of the Q and R matrices to obtain the global optimal control effect and has practical application value.
(3) Through the analysis of calculation and simulation, it can be seen that changing the unit coverage position can play a good role in suppressing the system vibration, especially in reducing the maximum amplitude of the system, but the convergence effect is not obvious. When the boundary condition is CFCF of the composite sandwich panel, the best coverage of the sandwich panel is near the solid support end (1, 2) unit. When the boundary condition is CFFF, the best coverage of the sandwich panel is near the solid support end (1, 2, 7, 8) units.
(4) The thickness of the damping layer affects the stability of the system after the failure of active control, and an appropriate increase in the thickness of the damping layer can enhance the passive control effect of the sandwich composite plates. The advantages of this method are a good suppression effect in the high-frequency vibration region and low control cost. The disadvantage is that the control effect is limited, the effect on the low-frequency region of the system vibration is not obvious, and the one-sided increase in thickness will lead to the overall mass of the system being too large. Therefore, the choice needs to be made according to the designer’s needs and the specific use scenario.

## Figures and Tables

**Table 1:** Comparison of Natural Frequencies of base under Different Boundary Conditions.

Mode	CFFF				CFCF			CCCC			
(HZ)	MATLAB	ANSYS	[21]	Test	MATLAB	ANSYS	Test	MATLAB	ANSYS	Test	Error
1	20.704	20.5635	20.446	20.312	134.55	132.4372	131.571	572.73	569.21	587.02	0.5%
2	89.305	87.1448	88.778	88.753	216.79	209.4320	210.242	704.08	691.26	698.61	1.2%
3	130.77	125.620	126.54	127.52	373.48	362.7831	368.721	1029.5	1010.53	1021.4	2.0%
4	291.06	281.926	283.41	284.25	491.15	479.9264	482.414	1571.3	1534.64	1565.7	3.2%
5	368.78	354.211	357.14	358.24	634.85	621.2412	628.457	2310.2	2284.41	2301.8	4.4%

**Table 2:** Natural Frequencies of Sandwich plate Element Covered in Order under Two Boundary Conditions.

Mode	Not Covered		Cover 1–2 Units		Cover 1–4 Units		Cover 1–6 Units		Cover 1–8 Units	
(HZ)	CFFF	CFCF	CFFF	CFCF	CFFF	CFCF	CFFF	CFCF	CFFF	CFCF
1	20.704	134.55	20.912	132.04	20.620	109.82	18.676	96.811	15.229	96.135
2	89.305	216.79	89.361	211.86	84.974	176.44	74.891	156.46	64.773	155.11
3	130.77	373.48	128.58	344.70	110.05	318.16	104.31	277.80	93.775	266.59
4	291.06	491.15	280.54	450.47	239.99	419.02	235.78	365.94	208.01	350.58
5	368.78	634.85	340.74	610.48	313.75	504.63	284.07	459.06	263.43	453.34

**Table 3:** Judgment indexes of controllability and observability before and after model reduction.

	Size (WC)	Rank (WC)	Size (WO)	Rank (WO)
Full model	168	72	168	81
Dynamic condensation	92	46	92	51
State-space	10	10	10	10

Note: Size () represents the matrix dimension, Rank () represents the rank of the matrix, WC represents the controllability matrix, and WO represents the observability matrix.

## Data Availability

Data sharing not applicable.

## References

[1] Huang Z., Qin Z., Chu F. (2019). A compression shear mixed finite element model for vibration and damping analysis of viscoelastic sandwich structures. J. Sandw. Struct. Mater..

[2] Huang Z., Wang X., Wu N., Chu F., Luo J. (2020). TheFinite Element Modeling and Experimental Study of Sandwich Plates with Frequency-Dependent Viscoelastic Material Model. Materials.

[3] Kumar A., Behera R.K. (2019). Passive Constrained Layer Damping: A State of the Art Review. IOP Conf. Ser. Mater. Sci. Eng..

[4] Zhu R., Zhang X., Zhang S., Dai Q., Qin Z., Chu F. (2022). Modeling and topology optimization of cylindrical shells with partial CLD treatment. Int. J. Mech. Sci..

[5] El Hafidi A., Herrero C.D.L.P., Martin B. (2015). Optimization of passive constrained layer damping (PCLD) treatments for vibration reduction. J. Vibroeng..

[6] Zhang L., Zhang F., Qin Z., Han Q., Wang T., Chu F. (2022). Piezoelectric energy harvester for rolling bearings with capability of self-powered condition monitoring. Energy.

[7] Kwak S.-K., Washington G., Yedavalli R.K. (2002). Acceleration Feedback-Based Active and Passive Vibration Control of Landing Gear Components. J. Aerosp. Eng..

[8] Baz A. (1997). Boundary Control of Beams Using Active Constrained Layer Damping. J. Vib. Acoust..

[9] Lam M.J., Inman D.J., Saunders W.R. (1997). Vibration Control through Passive Constrained Layer Damping and Active Control. J. Intell. Mater. Syst. Struct..

[10] Liu T., Hua H., Zhang Z. (2004). Robust control of plate vibration via active constrained layer damping. Thin-Walled Struct..

[11] Ray M.C., Oh J., Baz A. (2001). Active constrained layer damping of thin cylindrical shells. J. Sound Vib..

[12] Kattimani S.C., Ray M.C. (2018). Vibration control of multiferroic fibrous composite plates using active constrained layer damping. Mech. Syst. Signal Process..

[13] Baz A. (1997). Dynamic Boundary Control of Beams Using Active Constrained Layer Damping. Mech. Syst. Signal Process..

[14] Liao W., Wang K. (1996). Analysis and design of viscoelastic materials for active constrained layer damping treatments. Proc. SPIE.

[15] Shen I.Y. (1994). Bending-vibration control of composite and isotropic plates through intelligent constrained-layer treatments. Smart Mater. Struct..

[16] Huang Z., Qin Z., Chu F. (2016). Vibration and damping characteristics of sandwich plates with viscoelastic core. J. Vib. Control..

[17] Zhang C., Wang G., Wei D., Tian Y., Yang L. (2022). The research on the transverse vibration active control model of ship propulsion shaft with the active control force on the bearing support. Ocean Eng..

[18] Huang Z., Mao Y., Dai A., Han M., Wang X., Chu F. (2022). Active Vibration Control of Piezoelectric Sandwich Plates. Materials.

[19] Cao X., Tanner G., Chronopoulos D. (2020). Active vibration control of thin constrained composite damping plates with double piezoelectric layers. Wave Motion.

[20] Li M., Sun W., Liu Y., Ma H. (2022). Influence analysis of control signal phase on the vibration reduction effect of active constrained layer damping. Appl. Acoust..

[21] Lu P., Wang P., Lu J. (2021). Decentralized vibration control of smart constrained layer damping plate. J. Vib. Control..

[22] Cao Y.Q., Deng Z.X., Wang P. (2012). A Mechanics Model and Active Control for Smart Constrained Layer Damping Structure. Appl. Mech. Mater..

[23] Zhang D., Zheng L. (2014). Active Vibration Control of Plate Partly Treated with ACLD Using Hybrid Control. Int. J. Aerosp. Eng..

[24] Zheng L., Zhang D., Wang Y. (2011). Vibration and damping characteristics of cylindrical shells with active constrained layer damping treatments. Smart Mater. Struct..

[25] Lu J., Wang P., Zhan Z. (2017). Active vibration control of thin-plate structures with partial SCLD treatment. Mech. Syst. Signal Process..

[26] Li W., Yang Z., Li K., Wang W. (2021). Hybrid feedback PID-FxLMS algorithm for active vibration control of cantilever beam with piezoelectric stack actuator. J. Sound Vib..

[27] Gupta A., Panda S. (2021). Hybrid damping treatment of a layered beam using a particle-filled viscoelastic composite layer. Compos. Struct..

[28] Huang Z., Pan J., Yang Z., Wang X., Chu F. (2021). Transverse Vibration of Viscoelastic Sandwich Structures: Finite Element Modeling and Experimental Study. Materials.

[29] Li L., Zhang D., Guo Y. (2017). Dynamic modeling and analysis of a rotating flexible beam with smart ACLD treatment. Compos. Part B: Eng..

[30] Lu J., Zhan Z., Liu X., Wang P. (2018). Numerical modeling and model updating for smart laminated structures with viscoelastic damping. Smart Mater. Struct..

[31] Cao Y.Q. (2011). Study on Vibration and Noise Control for Car Body Structure Based on Smart Constrained Layer Damping. Ph.D. Thesis.

[32] Zhang D.D. (2011). Research on Multi-Objective Optimization and Adaptive Control of Damping Structures with Active Restraint Layer. Ph.D. Thesis.

[33] Lepoittevin G., Kress G. (2010). Optimization of segmented constrained layer damping with mathematical programming using strain energy analysis and modal data. Mater. Des..

[34] Felippe W., Barbosa F. (2017). A nondeterministic GHM based model applied to sandwich beams. Procedia Eng..

[35] Qu Z.-Q. (1998). A Multi-Step Method for Matrix Condensation of Finite Element Models. J. Sound Vib..

[36] Kamil H.G., Makki O.T., Umran H.M. (2020). Optimal tuning of a Linear Quadratic Regulator for Position Control using Particle Swarm Optimisation. IOP Conf. Ser. Mater. Sci. Eng..

[37] Liu Y., Qin Z., Chu F. (2021). Nonlinear forced vibrations of functionally graded piezoelectric cylindrical shells under electric-thermo-mechanical loads. Int. J. Mech. Sci..

[38] Ray M., Reddy J. (2013). Active damping of laminated cylindrical shells conveying fluid using 1–3 piezoelectric composites. Compos. Struct..

[39] Shah P.H., Ray M.C. (2013). Active Structural-Acoustic Control of Laminated Composite Truncated Conical Shells Using Smart Damping Treatment. J. Vib. Acoust..

[40] Mohammed H.A.U.Q., Wasmi H.R. (2018). Active Vibration Control of Cantilever Beam by Using Optimal LQR Controller. J. Eng..

[41] Tian J., Guo Q., Shi G. (2020). Laminated piezoelectric beam element for dynamic analysis of piezolaminated smart beams and GA-based LQR active vibration control. Compos. Struct..

[42] Ezzraimi M., Tiberkak R., Melbous A., Rechak S. (2018). LQR and PID Algorithms for Vibration Control of Piezoelectric Composite Plates. Mechanika.

[43] Mastali M., Kheyroddin A., Samali B., Vahdani R. (2016). Optimal placement of active braces by using PSO algorithm in near- and far-field earthquakes. Int. J. Adv. Struct. Eng..

